# Senior Health Clinic for 75-year-old home-dwelling Finns – study design, clinic protocol and non-response analysis

**DOI:** 10.1186/s12913-023-09199-9

**Published:** 2023-03-02

**Authors:** Marika Salminen, Sari Stenholm, Jaana Koskenniemi, Päivi Korhonen, Tiina Pitkänen, Paula Viikari, Maarit Wuorela, Matti Viitanen, Laura Viikari

**Affiliations:** 1 Turku University Hospital/Medical domain, Wellbeing services county of Southwest Finland, Turku, FIN-20521 Turku Finland; 2 Faculty of Medicine, Department of General Practice, University of Turku, Joukahaisenkatu 3-5 A, Turku, 20014 Finland; 3 Department of Public Health, Faculty of Medicine, University of Turku and Turku University Hospital, 20014 Turku, Finland; 4 Centre for Population Health Research, University of Turku and Turku University Hospital, 20014 Turku, Finland; 5 Faculty of Medicine, Department of Geriatric Medicine, University of Turku and Turku University Hospital, Kunnallissairaalantie 20, Turku, 20700 Finland; 6 Division of Clinical Geriatrics, NVS, Karolinska Institutet, Karolinska University Hospital, Huddinge, 14186 Stockholm, Sweden

**Keywords:** Health clinic, Home-dwelling, Non-response, Older people

## Abstract

**Background:**

In the Finnish policy on older people preventive activities, which maintain functional capacity and independent living, are emphasized. The Turku Senior Health Clinic, aimed at maintaining independent coping of all home-dwelling 75-year-old citizens in the city of Turku, was founded in the beginning of 2020. The aim of this paper is to describe design and protocol of the Turku Senior Health Clinic Study (TSHeC) and provide results of the non-response analysis.

**Methods:**

The non-response analysis used data from 1296 participants (71% of those eligible) and 164 non-participants of the study. Sociodemographic, health status, psychosocial and physical functional ability indicators were included in the analysis. Participants and non-participants were also compared in respect to their neighborhood socioeconomic disadvantage. Differences between participants and non-participants were tested using the Chi squared or Fisher´s exact test for categorical variables and t-test for continuous variable.

**Results:**

The proportions of women (43% vs. 61%) and of those with only satisfying, poor or very poor self-rated financial status (38% vs. 49%) were significantly lower in non-participants than in participants. Comparison of the non-participants and participants in respect to their neighborhood socioeconomic disadvantage showed no differences. The prevalence of hypertension (66% vs. 54%), chronic lung disease (20% vs. 11%), and kidney failure (6% vs. 3%) were higher among non-participants compared to participants. Feelings of loneliness were less frequent among non-participants (14%) compared to participants (32%). The proportions of those using assistive mobility devices (18% vs. 8%) as well as those having previous falls (12% vs. 5%) were higher in non-participants than in participants.

**Conclusions:**

The participation rate of TSHeC was high. No neighborhood differences in participation were found. Health status and physical functioning of non-participants seemed to be slightly worse than those of the participants, and more women than men participated. These differences may weaken the generalizability of the findings of the study. The differences have to be taken into account when recommendation for the content and implementation of preventive nurse-managed health clinic in primary health care in Finland is going to be given.

**Trial registration:**

ClinicalTrials.gov Identifier: NCT05634239; registration date; 1st of December 2022. Retrospectively registered.

## Background

The Finnish policy on older people emphasizes the priority of living at home. Because the number of older people continues to grow, the number of those with morbidity, comorbidity and/or frailty is also growing. Thus, preventive activities, which maintain functional capacity and independent living, are highly emphasized [1].

Both cardiovascular diseases (CVDs) and dementia are highly prevalent among older people, and they share several modifiable risk factors supporting the possibility of preventive interventions [2, 3]. In people aged 75 or older, leading vascular metabolic risk factors are high systolic blood pressure, high fasting plasma glucose, diabetes, high body mass index and high LDL cholesterol [3, 4]. Metabolic risks are increasing, on average, every year, which means that no real progress in reducing behavioral risks has been achieved. Combination of aging population and increasing metabolic risks most likely maintains the increasing trends in non-communicable diseases [5]. Moreover, CVDs, impaired cognitive function and dementia are associated with a considerably increased risk of disability [3, 6–8], hospitalization [9, 10], institutionalization [11–13], and/or mortality [6, 9, 14].

Frailty is common among older people, especially among older women, even though the prevalence of frailty varies according to the measurement used [15–18]. Frailty has shown to be highly common in older people with CVDs [16, 19], and it worsens prognosis of the CVD patients [6, 9, 16]. Frailty is also strongly associated with dementia, cognitive impairment [8, 20] and multimorbidity [21]. Frailty increases the risk of hospitalization [22], institutionalization [23] and mortality [13, 15, 22]. In addition to metabolic risk factors, CVDs, cognitive impairment, dementia, and frailty, also pain [24], musculoskeletal conditions [25, 26], depression [26] and loneliness [27] may threaten functional ability and independent coping of older people and, thus, should be screened for preventive actions.

The key themes in the Finnish national recommendation to guarantee a good quality of life and improved services for older persons include promoting the functional capacity of older people, increasing voluntary work, utilizing digitalization and technologies, organizing and providing services, arranging guidance and service coordination, ensuring skilled personnel the quality of services [1]. During the past 10 years, various preventive health clinics for older people have already been implemented in municipalities and cities in Finland. However, to the best of our knowledge, no systematic assessment of the findings, applicability and/or effects of these procedures have been implemented so far.

The main purpose of this paper is to describe the study design and protocol of the Turku Senior Health Clinic Study (TSHeC). Results of the non-response analysis are also provided.

## Material and methods

### Study population

**Fig. 1:**
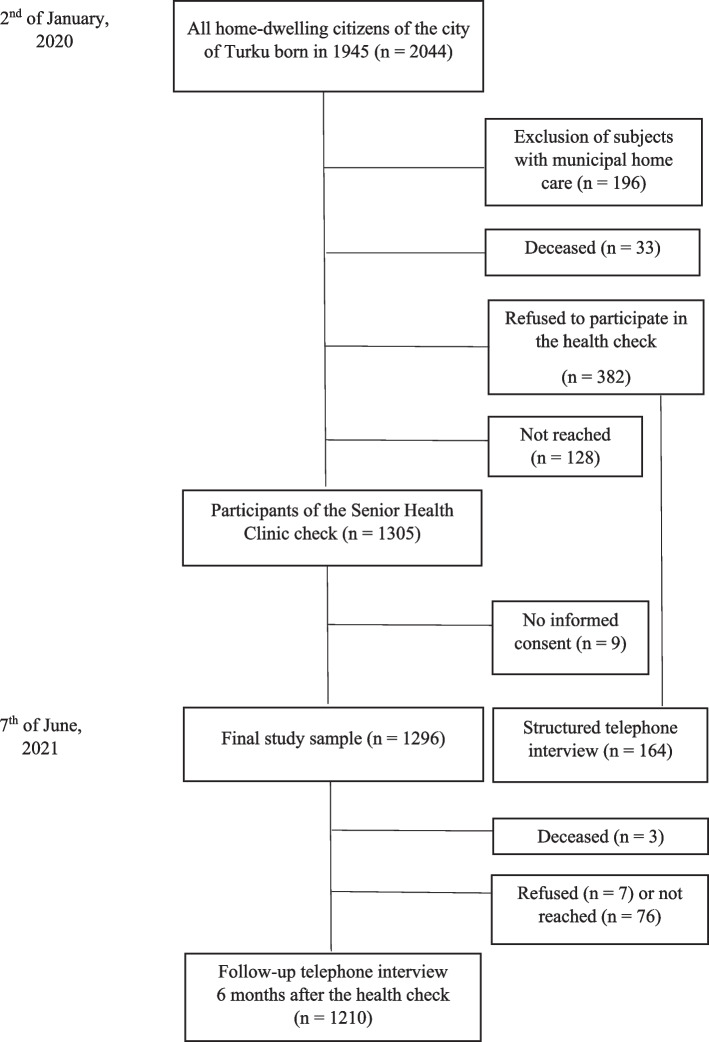
Flow chart of the study

TSHeC population consisted of all Finnish and Swedish speaking home-dwelling citizens born in 1945 in the city of Turku, in southwestern Finland in the beginning of 2020 (*n* = 2044). Those with municipal home care (*n* = 196) were excluded from the study population, 33 deceased before invitation, 382 refused to participate in the clinic´s health check, and 128 were not reached. Of those 1305 examined at the clinic, nine subjects declined to participate in the study, leaving 1296 study participants (71% of those eligible). The flow chart of the study is shown in Fig. 1.

The study sample in non-response analysis included 1296 study participants examined at the clinic and 164 subjects who refused to participate (43% of 382 subjects who refused to participate) in the health clinic check but were willing to answers a short, structured telephone interview.

### Turku Senior Health Clinic Study

TSHeC was targeted at 75-year-old citizens with an underlying idea that at that age it would be almost the last moment to nudge people towards healthy lifestyle and taking care of their health and functional ability in order to maintain living independently.

The short-term aims of TSHeC are to survey health and functional statuses, and prevalence of specified risk factors for CVDs, dementia, frailty, and functional decline of 75-year-old independently home-dwelling citizens of the city of Turku. The aims also include assessment of the frequency of follow-up treatments needed, and recommendations given for lifestyle changes and evidence-based use of medication, as well as enforcement of these recommendations. Also, participants´ feedback on TSHeC will be assessed. In addition, based on the results, recommendations considering the protocol and implementation of preventive health clinics targeted at older people, will be provided. The long-term aim of the research project is to assess the effects of the TSHeC on the need of institutional care and home care provided by the city of Turku as well as the cost-effectiveness of the clinic during the 10-year follow-up. For this purpose, participants of TSHeC will be compared to non-participants and earlier cohorts of 75-year-olds in terms of the use of home care and institutional care.

### Recruitment

Before the clinic was founded, a couple of media articles about the upcoming, free of charge, health check were published in the local newspaper. Contact information of all home-dwelling citizens of the city of Turku born in 1945 was requested from the Finnish Digital and Population Data Services Agency. A clinic nurse contacted eligible subjects by phone. During the phone call, subjects were given information on TSHeC. A written invitation was sent to those who were not reached by phone. After receiving the written invitation, they were reached again by phone, twice, if needed. Those who declined to participate in the health check were encouraged to at least participate in a structured telephone interview. Those who refused were not contacted again. The personnel of the health clinic was bilingual, which eased the participation of Swedish-speaking subjects.

### Clinic protocol and data collection

**Fig. 2:**
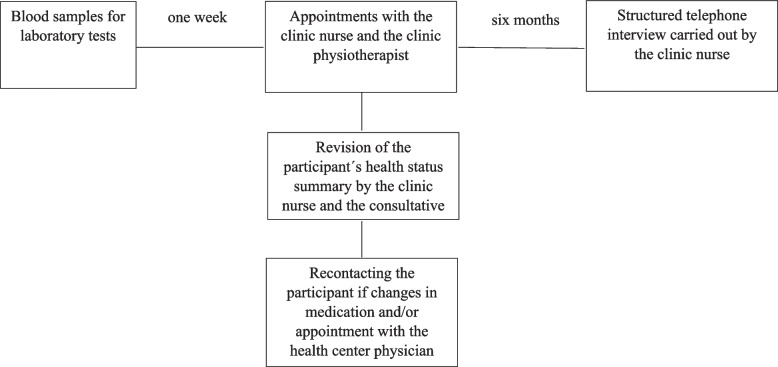
Senior Health Clinic protocol

TSHeC for 75-year-old independent home dwellers was implemented between January 2020 and June 2021 in the Turku City Hospital by three trained clinic nurses, two physiotherapists, and a consultative geriatrician. Appointments to health checks were scheduled to those willing to participate. They were sent written information and postal questionnaire concerning their sociodemographic, health behavior, health status, psychosocial and physical functional ability (Table 1). They were advised to take the filled questionnaire along to the appointments. Blood samples were drawn one week before the clinic appointments at the units of the Turku University Hospital laboratory (Tykslab) and analyzed at Tykslab. Clinic protocol is demonstrated in Fig. 2.

**Table 1:** Content of the Turku Senior Health Clinic check

	**Postal questionnaire**	**Nurse´s appointment**	**Physiotherapist´s appointment**
Sociodemographics	gender marital status living situation education economic status		
Health behaviour	smoking alcohol use circadian rhythm frequency of exercise		
Health status	self-rated health diagnosed diseases continence use of medication	Interview - pain - fatigue/tiredness - mood - cognition (6CIT^a^) - fracture risk (FRAX^b^) - risk for diabetes - oral health Clinical examination - height - weight - waist and hip circumferences - vision - hearing - blood pressure - pulse - orthostatic hypotension	
Psychosocial functional ability	quality of life satisfaction loneliness social participation hobbies		
Physical functional ability	need for help in everyday living instrumental activities of everyday living physical activity recent health related changes in physical activity fear of falling		Interview - use of assistive mobility device - managing in everyday living (walking 400 m, climbing stairs, doing housework, using public transportation vehicle, cutting toenails) - fear of falling (FES-I^c^) - falls risk (FROP-Com Screen^d^) Physical examination - hand grip strength - balance, walking speed, sit to stand (SPPB^e^) - one-leg stand - 30 s sit-to-stand - bening paroxysmal positional vertigo
Laboratory tests (1 week before nurse´s appointment)		- electrocardiogram - complete blood count - creatinine - alanine transaminase - glucose - thyreotropin - vitamin B12 - folate - total, HDL and LDL cholesterol, triglyserides - calcium - vitamin D	

^a^Six Item Cognitive Impairment Test [28]

^b^Fracture Risk Assessment Tool [29]

^c^Falls Efficacy Scale—International [30]

^d^Three items of the Falls Risk for Older People in the Community tool [31]

^e^Short Physical Performance Battery [32]

During the 60–90-min appointment with the clinic nurse, results of the laboratory tests and the questionnaire filled beforehand were reviewed together with the participant. More information on the participant´s health status was gathered with an interview, and the participant was clinically examined by the clinic nurse. Participant´s health issues were discussed. If there was a need for and/or a possibility to lifestyle changes (e.g., in diet, exercising, social activation, weight control), the clinic nurse encouraged the participant to make those changes. After the appointment, the clinic nurse made a summary of each participant´s health status, which was reviewed together with the consultative geriatrician. The clinical nurse contacted the participant again if changes in medication and/or an appointment with health center physician was suggested by the geriatrician.

Appointment with the physiotherapist lasted from 30 to 45 min and included the assessment of physical functioning, the use of assistive mobility devices, and managing in everyday living. During the appointment, participants got individualized information on physical training and nutrition, especially protein intake, to maintain and/or improve their physical functioning. The content of the appointment was highly preventive and supportive, and information and suggestions given were based on participant´s level of physical functioning, motivation, and own goals. For those interested, group exercises suitable for their needs, e.g., muscle strengthening and/or balance training, were suggested. Participants were encouraged to maintain independence in mobility without mobility devices by improving muscle strength and balance. However, if there was a need for mobility devices to ensure safety, an appointment to the Assistive Technology Services of the city of Turku was scheduled. In case of musculoskeletal conditions, participants got self-care advice and/or an appointment with a health center physician, if needed.

If participant had symptoms or diseases that needed urgent medical care, geriatrician of Urgent Geriatric Outpatient Clinic was immediately consulted. In non-urgent cases, an appointment with health center physician, dentist of Oral and Dental Care, coordinator of the Memory Clinic, psychiatric nurse and/or dietician was scheduled. In addition to municipal services, services of local voluntary third-sector organizations and expert institutions were also recommended, and appointments scheduled if there was a need for rehabilitation, social activities, housing services, care, and supportive services as well as health-promoting activities. All municipal follow-up treatment facilities, personnel, and collaborators as well as voluntary third-sector collaborators are shown in Table 2.

**Table 2:** Follow-up treatment facilities, personnel, and collaborators of the Senior Health Clinic

*Municipal services*
Urgent Geriatric Outpatient Clinic
Local Health Centers/Stations
Oral and Dental Care
Memory Clinic
Psychiatric nurse
Dietician
Service guidance for older people
Welfare centers for older people
Sport Services Centre
Strength in Old Age Program
Assistive Technology Services
*Voluntary third-sector collaborators*
Nine local registered voluntary organizations and expert institutions aimed to enhance psychosocial, mental, cognitive and physical functioning and quality of life of older people by organizing rehabilitation, social activities, housing services, care and supportive services as well as health-promoting activities for older people and their care givers

Six months after the TSHeC check, the clinic nurse contacted participants for a structured follow-up telephone interview. During the 10–20-min interview, participants were asked how they experienced the clinic and the content of the clinic protocol, and about the responsiveness of the clinic protocol to their needs, and adherence to different changes (concerning medication, diet, exercising, weight control, and/or social activity) they were encouraged to make, if there were any.

### Ethics

The study was conducted according to the guidelines of the Declaration of Helsinki. The Ethics Committee of the Hospital District of Southwest Finland approved the study protocol (Diary number 87/1801/2019). Participants provided written informed consent for the study.

### Statistical analyses

The present analyses used data of 1296 participants of the TSHeC check and 164 non-participants who were willing to participate in a short, structured telephone interview. For the non-response analysis, we included sociodemographic, health status, psychosocial and physical functional ability indicators. In addition, we compared participants and non-participants in respect to their neighborhood socioeconomic disadvantage. By using postal code information from the Statistics of Finland, standardized index of neighborhood socioeconomic disadvantage was calculated by using median household income in 2020 (coded as additive inverse), low educational attainment in 2020 (percentage of people over 18 years old with low education) and unemployment rate in 2019 (unemployed people belonging to the labor force/total labor force) [33]. For each of the three variables, we derived a standardized z score (national mean = 0, Standard deviation = 1). A total disadvantage score was then calculated by taking the mean value across all z scores; the mean of the score in the study population was 0.05 (range − 1.54 to 2.16), with a higher score indicating a higher disadvantage.

Differences between participants and non-participants were tested using the Chi squared or Fisher´s exact test for categorical variables and two-sample t-test for a continuous variable. *P* values less than 0.05 were considered statistically significant. All statistical analyses were performed using SAS System for Windows, version 9.4 (SAS Institute INC., Cary, NC, USA).

## Results

The proportions of women (43% vs. 61%) and of those with only satisfying, poor or very poor self-rated financial status (38% vs. 49%) were significantly lower in non-participants than those of participants were. Comparison of the non-participants and participants in respect to their neighborhood socioeconomic disadvantage, by using the data from the Statistics of Finland, showed no differences between the two groups. Of 13 diseases or chronic conditions that had possibly been previously diagnosed, significant differences between the non-participants and participants were found only in the prevalence of hypertension (66% vs. 55%), chronic lung disease (22% vs. 11%), and kidney failure (6% vs. 3%), all being higher among the non-participants. Feelings of loneliness were significantly less frequent among the non-participants (14%) compared to the participants (32%). The proportions of those using assistive mobility devices (18% vs. 8%) as well as those having falls during the previous 12 months (12% vs. 5%) were significantly higher in non-participants compared to the participants (Table 3).

**Table 3:** Characteristics of participants and non-participants of the Turku Senior Health Clinic Study

	Participants (*n* = 1296) n (%)	Non-participants (*n* = 164) n (%)	*P*-value^a^
Female	789 (61)	71 (43)	** < 0.001**
Living alone	483 (37)	60 (37)	0.865
Neighborhood socioeconomic disadvantage index, mean (SD)	0.27 (0.83)	0.29 (0.82)	0.831
Education			0.124
University	248 (19)	23 (14)	
Post-secondary level or university of applied sciences	284 (22)	30 (18)	
Vocational upper or general secondary education	329 (25)	53 (32)	
Basic education or none	434 (34)	58 (35)	
Self-rated financial status			**0.013**
Very good or good	668 (52)	101 (62)	
Satisfying	556 (44)	60 (37)	
Poor or very poor	62 (5)	2 (1)	
Having someone who helps when needed			0.054
Yes	574 (45)	67 (41)	
No	45 (4)	1 (1)	
No need for help	656 (51)	96 (59)	
Self-rated health			0.311
Very good or good	666 (51)	77 (47)	
Moderate	528 (41)	69 (42)	
Poor or very poor	102 (8)	18 (11)	
Diabetes	247 (20)	38 (23)	0.304
Coronary artery disease	143 (12)	21 (13)	0.641
Myocardial infarction	71 (6)	10 (6)	0.848
Heart failure	86 (7)	14 (9)	0.453
Hypertension	666 (54)	108 (66)	**0.002**
Stroke or transient ischemic attack	112 (9)	16 (10)	0.770
Cancer	270 (22)	36 (22)	0.904
Chronic lung disease	135 (11)	32 (20)	**0.001**
Rheumatoid arthritis or osteoarthritis	288 (24)	30 (18)	0.121
Kidney failure	33 (3)	10 (6)	**0.016**
Parkinson´s disease	17 (1)	1 (1)	0.712
Mental disease	52 (4)	6 (4)	0.743
Other chronic disease	326 (28)	52 (32)	0.366
Feelings of loneliness			** < 0.001**
Not at all	890 (69)	142 (87)	
Sometimes	384 (30)	21 (13)	
Often or always	21 (2)	1 (1)	
Depressive symptoms during the previous month	143 (11)	19 (12)	0.840
Feelings of fatigue during the previous month			0.109
Not at all	837 (65)	101 (62)	
Sometimes	332 (26)	53 (32)	
Often–all the time	122 (9)	10 (6)	
Assistive mobility device	109 (8)	29 (18)	** < 0.001**
Self-rated ability to walk 400 m	1254 (97)	157 (96)	0.644
Self-rated ability to climb stair one floor at one go	1268 (98)	156 (96)	0.070
Number of falls during the previous 12 months			**0.003**
None	1221 (95)	144 (88)	
1–2	41 (3)	18 (8)	
≥ 3	29 (2)	7 (4)	
Reduction of daily exercising during the previous 12 months	299 (23)	46 (28)	0.167

^a^X^2^-test or Fisher´s exact test for categorical and T-test for continuous variables

Reasons for non-participation were not systematically documented, and only a part of the non-participants explained their reasons for non-participation. The most frequently mentioned reasons for non-participation were regular medical controls due to a chronic condition, regular health checks in private health care, and ongoing care of a severe illness. Fear of COVID-19 was mentioned only a few times.

## Discussion

All 75-year-old home-dwelling citizens in the city of Turku, who did not have municipal home care, were invited to the TSHeC. Intensive efforts were made to increase the response rate before and during the recruitment phase. During the survey preparation, media articles were published in the local newspaper and eligible subjects were contacted several times, if needed. Bilingual personnel of the health clinic eased the participation of Swedish-speaking subjects. With these evidence-based recruitment strategies [34], participation rate of 71%, that is consistent with those of other population-based Finnish studies among older people [12, 26], was achieved.

The protocol of TSHeC was implemented between January 2020 and June 2021. Due to the COVID-19 pandemic, a four and a half month´s break in health clinic appointments was held between March and August 2020. Although not all non-participants indicated reasons for non-participation, fear of COVID-19 was mentioned only a few times. However, non-participants of the Turku Senior Health Clinic Study were more likely to be male, less likely to suffer from feelings of loneliness and/or they had better self-rated financial status than the participants of the study. Factors associated with non-participation were earlier studied in Japanese population-based cohort study aimed to prevent lifestyle-related diseases [35], in a survey of Norwegian coronary heart disease patients [36], in a randomized controlled trial of multidomain lifestyle intervention for prevention of cognitive decline among French dementia-free subjects [37], and in a review article exploring characteristics of those who do and do not engage with preventive health checks [38]. In most earlier studies, non-participants were more likely women [35–37] and had lower socioeconomical statuses, which are inconsistent with the results of the present study [35, 37, 38]. Only according to the review article [38], non-participants were more likely men, as in the present study. It is to be noted, that most of the earlier studies were conducted among subjects of all-ages, also including subjects under the age of 65 years [35, 36, 38].

Because of the existing evidence of the association of socioeconomic status and health behavior, functional ability, health, as well as mortality in older people [39–41], the index of neighborhood socioeconomic disadvantage was added in non-response analyses. The aim was to examine if there are challenges in participation and, due to this, a need for targeted specific strategies for preventive actions in certain neighborhoods. The results of this study showed no difference in the neighborhood socioeconomic disadvantage level between the participants and non-participants, which suggests that people were willing and able to participate across the city of Turku. However, consistent with earlier studies [36, 42, 43], health status and physical functional ability of the non-participants seemed to be slightly worse than those of the participants. Non-participants have shown to value health less strongly, have lower interest in obtaining personal health information, have low self-efficacy, feel less in control of their health, prefer less time-demanding studies, and are less likely to believe in the efficacy of health checks [38, 43]. Found differences between participants and non-participants may deteriorate the generalizability of the findings of our study, and, have to be taken into account when recommendation for the content and implementation of preventive nurse-managed health clinic in primary health care in Finland is going to be given.

Because of the prevalence of frailty [15–18] and its associations to morbidity [6, 8, 9, 16, 19, 20], hospitalization [22], institutionalization [23] and mortality [15, 18, 22] among older people, preventive and supportive appointment with a physiotherapist was also included in the content of the TSHeC. All participants got the assessment of physical functional ability and individualized information to maintain and/or improve physical functioning and independent mobility by the clinic physiotherapist, not only those with a clear need for it.

Several chronic diseases and geriatric syndromes have overlapping risks and protective factors [44]. The aims of the nurse-managed TSHeC are to examine the health, functional ability, and risk factors of home-dwelling 75-year-olds and by tackling these risk factors, diminish adverse outcomes such as decline in functional ability, institutionalization, and death. Evidence exists that nurse-managed preventive health clinics for older adults with medical, functional and health behavior components improve the access to care, use of preventive services, support in the promotion of health, management of chronic diseases, adherence to treatment and patient satisfaction, patient outcomes, and reduce hospitalization [45–47]. By the structured telephone interview six months after the health check, the adherence to different activities suggested can be assessed.

The strength of this study is a large sample size which e.g., enables us to determine subgroups with best adherence for different suggested health behavior changes. Also, some limitations must be mentioned. Reasons for non-participation were not systematically recorded. Another limitation is the low response rate of the non-participants; less than half of the non-participants were willing to participate a short, structured telephone interview. It is possible that non-participants who refused to answer even a short telephone interview had worse health status and physical functional ability compared to those who were willing to answer. This may have biased the data of non-participants.

Both by the nurturing and supportive health check and by the six-month follow-up telephone interview, participants were nudged towards a healthy lifestyle, taking care of their health and functional ability to maintain independent coping. TSHeC will add knowledge about the health and functional ability of community-dwelling older adults as well as adherence to recommended health behavior changes. Based on these results of the TSHeC, recommendations for the protocol and implementation of preventive nurse-managed health clinics in primary health care in Finland is aimed to be given to support more homogeneous preventive service for older people in Finland.

## Data Availability

The data used and/or analyzed during the current study is available from the corresponding author by reasonable request.

## Abbreviations

CVD: Cardiovascular disease
LDL: Low-density lipoprotein
TSHeC: Turku Senior Health Clinic Study
Tykslab: Turku University Hospital laboratory
